# Structure-based inhibitory peptide design targeting peptide-substrate binding site in EGFR tyrosine kinase

**DOI:** 10.1371/journal.pone.0217031

**Published:** 2019-05-22

**Authors:** Farial Tavakoli, Mohamad Reza Ganjalikhany

**Affiliations:** Department of Biology, Faculty of Sciences, University of Isfahan, Isfahan, Iran; Hungarian Academy of Sciences, HUNGARY

## Abstract

EGFR (epidermal growth factor receptor) plays the critical roles in the vital cell activities, proliferation, differentiation, migration and survival in response to polypeptide growth factor ligands. Aberrant activation of this receptor has been demonstrated in many human cancers, particularly in non-small cell lung carcinoma (NSCLC). L858R point mutation is the most common oncogenic mutation in EGFR tyrosine kinase domain in patients with EGFR-mutated NSCLC. A feedback inhibitor of EGFR is MIG6 molecule which binds peptide-substrate binding site of the receptor and leads to degradation of activated EGFR. In this *in silico* study, the peptide-substrate binding site of EGFRL858R mutant has been targeted to inhibit it using molecular docking, MD simulation and MM-PBSA method. Finally, physicochemical properties of the designed peptides have been evaluated. A peptide library was provided composed of 31 peptides which were designed based on the MIG6 structure. The results indicated that, two peptides were able to inhibit EGFRL858R mutant selectively. This computational study could be helpful in designing novel inhibitory peptides to inhibit oncogenic EGFR mutants which do not respond to available EGFR TKIs.

## Introduction

The human epidermal growth factor receptor (EGFR) family that contains four closely related receptors (EGFR/ErbB1/HER1, ErbB2/HER2, ErbB3/HER3, and ErbB4/HER4) plays pivotal roles in the regulation of cell proliferation, differentiation, migration, and survival in response to polypeptide growth factor ligands. Overexpression or mutations of EGFR has been demonstrated in tumor cell formation and proliferation in some of human cancers such as liver, breast, stomach, colorectal cancers and particularly in glioblastoma and non-small cell lung carcinoma (NSCLC) [1–4]. Structurally, EGFR is composed of an extracellular ligand receptor, a transmembrane region, and an intracellular tyrosine kinase domain. Once ligand binds the receptor, EGFR activates and forms an asymmetric dimer in tyrosine kinase domain, in which the C-lobe of the kinase domain (activator EGFR) contacts to the N-lobe of the receiver EGFR and stabilizes it in the active state conformation. Active EGFR leads to signaling process through RAS-MAP kinase, PI3K-AKT and other pathways [1, 5].

Before activation, the kinase is in an autoinhibited state by a feedback inhibitor called MIG6 (an encoded cytosolic protein by mitogen-inducible gene6). EGFR signalling induces MIG6 expression through the RAS-MAP kinase pathway and also activates Src. Src primes MIG6 molecule to bind EGFR via phosphosrylation of Tyr 395, then MIG6 binds EGFR and is double phosphorylated on Tyr 394 that leads to inhibit EGFR.

C-terminal region of MIG6 protein comprises 2 segments, segment 1 (residues 336–364) and segment 2 (residues 365–412) which includes a principal phosphorylation site, tyrosine residues (Tyr 394 and Tyr 395). This region plays the main role in binding and inhibition of EGFR tyrosine kinase by blocking the formation of an active dimer receptor [3, 6]. The data obtained from crystal structure of MIG6 segment 1+2 in complex with wild type EGFR (WT-EGFR) show that one MIG6 molecule binds receiver EGFR allosterically by blocking the asymmetric dimer interface through the segment 1 and occupies the peptide-substrate binding site via segment 2. In this way, the prior phosphorylated MIG6 on residue Tyr 395 (MIG6-YpY) via Src, directly and exclusively binds to activated EGFR peptide-substrate binding site and then is double phosphorylated on residue Tyr 394 (MIG6-pYpY) through activated EGFR [3, 7]. At this time, the MIG6-pYpY rearranges to form a hairpin-like element which leads to block the peptide substrate-binding cleft and inhibit EGFR downstream signaling pathway [3].

The peptide-substrate binding site is a shallow and solvent exposed cleft which includes two main pockets, phosphoacceptor and priming recognition pockets. Phosphoacceptor pocket as a significant pocket contains a catalytic base (Asp 837) and is responsible for the phosphorylation of the substrates on a tyrosine residue in P position of phosphorylation site. Priming recognition pocket includes Lys 879 and Ala 920 which is able to recognize the substrates that are primed by prior phosphorylation of a tyrosine residue in P+1 position [3, 6, 8, 9].

EGFR is one of the most studied receptor tyrosine kinases in the cancer drug target field [5, 10, 11]. Over 99% of EGFR tyrosine kinase inhibitors (TKIs) which have been reported to date, are ATP- competitive inhibitors and target the ATP-binding pocket in the catalytic domain of EGFR kinase [12]. Erlotinib and gefitinib as the first generation of EGFR TKIs and afatinib, neratinib and dacomitinib as the second generation of EGFR TKIs are reversible and irreversible ATP-competitive inhibitors, respectively which have not provided the survival benefits to the patients with EGFR-mutated NSCLC [4, 13–15]. AZD9291 (Osimertinib) as the latest ATP-competitive TKI is an oral, potent and mutant-selective inhibitor which inhibit EGFRT790M, acquired resistance mutation, with a higher response rate than the other mutants EGFR in an irreversibly manner [11].

Although many of these ATP-competitive TKIs are approved by FDA, there are some important disadvantages reported for these inhibitors. Since the ATP-binding site of EGFR is highly conserved among human EGFR family members, this leads to off-target binding and toxicity of these compounds. Additionally, these inhibitors have to compete with intracellular ATP concentration in milimolar range while the K_M_ value of kinases for ATP is in micromolar range, then the higher affinities in nanomolar to picomolar ranges is required for these inhibitors to inhibit EGFR *in vivo* [12]. Finally, acquired resistance mechanism which is resulted from the emergence of an additional EGFR mutation, T790M, after treatment initiation with the ATP-competitive TKIs makes the treatment process more complicated. EGFRT790M mutation failed the long-term efficacy of many ATP-competitive inhibitors via increasing the affinity of mutant EGFR for ATP [11, 12, 14, 16].

In order to avoid these drawbacks, the attention has been recently focused on peptide-substrate binding site to discover inhibitors to inhibit EGFR tyrosine kinase via inhibition of protein-protein interaction. In contrast to the ATP-binding pocket, the peptide-substrate binding site is less conserved across the EGF receptor tyrosine kinase family members that lead to improving the selectivity. In addition, the kinase substrates usually exhibit binding affinity at their K_M_ value or lower *in vitro*, therefor it is not required the inhibitors to have a high affinity *in vitro* activity [3, 12].

Over the recent years, peptide based therapeutics have been considered as a promising and novel approach to treat diseases, particularly cancers. Several favorable characteristics of inhibitory peptides including ease of synthesis and modification, low toxicity, biocompatibility and the higher target selectivity and potency make them effective alternatives to small chemical drugs [17–20].

Given that the peptide-substrate binding site is a shallow cleft with several residues covering a wide surface area of the site and is less conserved across the EGFR tyrosine kinase family members compared to ATP-binding site [3, 12]. Additionally, with respect to the disadvantages of TKIs, the inhibitory peptides are more suitable alternatives to small molecule inhibitors to cover this site, because of their larger molecular size. Indeed, peptides can be designed with enhanced specificity to target desired regions with higher selectivity and potency even at very low doses [17].

Among somatic mutations in the EGFR kinase domain including, deletions in exon 19 (Ex19Del), insertions in exon 20 (Ex20Ins) and point mutations L858R, T790M, G719X and L861Q, L858R in exon 21 is the widespread oncogenic mutation in this domain in patients with EGFR-mutated NSCLC [2, 3, 10, 13]. Moreover, L858R-mutant EGFR (EGFRL858R) enhances the catalytic activity as much as 20–50 fold than WT-EGFR [10, 21].

Since only the receiver subunit of WT-EGFR asymmetric dimer is active and contributes to EGFR signaling, thus, one single MIG6 molecule should be sufficient to inhibit the ligand-stimulated WT receptor. By contrast, both kinases in EGFRL858R are presumably active and contribute to EGFR signaling, therefore, both active kinases need to be inhibited. Given that MIG6 is not capable of accessing to the asymmetric dimer interface of the activator subunit and is not expressed sufficiently to bind both subunits, inhibitory peptides with higher affinity than MIG6 segment 2 seems to be effective in inhibiting the EGFRL858R [3].

Computer-aided drug design has been recently applied broadly as a useful tool in biochemical and pharmaceutical sciences. These techniques can significantly contribute to the drug discovery and development of lead compounds as well as reduction of the experimental time and cost which is necessary to devote in laboratories [22]. Molecular dynamics simulation and molecular docking are key tools that are applied broadly to build, visualize and analyze molecular structures and their structure-activity relationship at atomic level [23].

In this study, we have employed *in silico* techniques to target the peptide-substrate binding pocket by inhibitory peptides considering the structure of MIG6 segment 2, in order to inhibit EGFRL858R. Finally, physicochemical properties of the designed peptides were investigated.

## Material and methods

### 3D structural investigation

Crystal structures of EGFRL858R and WT-EGFR in complex with Mig6-pYpY were obtained from Protein Data Bank (PDB ID: 4R3R with resolution 3.25 Å and 4ZJV with resolution 2.7 Å). The ligand and water molecules were removed from the complexes. EGFR structures were in asymmetric homodimer form, so a monomer was used for the study. Swiss Model server was used in order to fix the chain breaks in the structure [24].

The crystal structures of EGFRL858R and WT-EGFR are composed of 323 residues and the peptide-substrate binding site of both receptors consists of residues Asp837, Arg841, Asn842, Asp855, Lys875, Val876, Pro877, Ile878, Lys879, Trp880, Ser885, Ala920 (equals to residues Asp 142, Arg 146, Asn 147, Asp 160, Arg 163, Lys 180, Val 181, Pro 182, Ile 183, Lys 184, Trp 185, Ala 225 of EGFRL858R modeled structure and Asp 145, Arg 149, Asn 150, Asp 163, Leu 166, Lys 183, Val 184, Pro 185, Ile 186, Lys 187, Trp 188 and Ala 228 of WT-EGFR modeled structure).

### Peptide library

A peptide library is provided comprising 31 peptides (S1 Table) which are derivatives of MIG6 segment 2 (residues Thr392, His393, Tyr394, Tyr395, Leu396, Leu397 and Pro398). Avogadro v.1.2.0 was used to prepare peptide structures [25].

### Docking studies

GRAMM-X protein-protein docking server (v.1.2.0) (http://vakser.compbio.ku.edu/resources/gramm/grammx) was used to perform peptides docking on the WT-EGFR and EGFRL858R [26]. Ten obtained output conformation models from each docking are visualized using Accelrys Discovery Studio 4.0 Visualizer (DS 4.0, Accelrys Software Inc., San Diego, CA).

Rosetta FlexPepDock server (http://flexpepdock.furmanlab.cs.huji.ac.il/) was used to perform local refinement of the best-estimated conformation of a peptide in complex with receptor using flexible side chain [27]. Rosetta FlexPepDock server uses the Rosetta algorithm for scoring complexes and predicting the energy of each complex in Rosetta Energy Unit [21]. The output for FlexPepDock is including ten predicted binding energies for each complex in REU. The binding pose with the lowest interaction score was analyzed using Discovery Studio and saved for next studies.

### Molecular dynamics simulation

In order to investigate the structural dynamics in free EGFR models and EGFR-peptide complexes and also calculate free binding energy of EGFRs in complex with peptides, GROMACS 2016.4 package was used to perform MD simulation [28]. The protonation state of His residues in both protein and ligand molecules was determined using PDB2PQR web server [29].

The required topology and coordinate files for the both proteins and peptides were generated using GROMACS [28] and AmberTools [30] packages using Amber ff99SB all-atom force field [31].

Each system was immersed in the center of a dodecahedron box of TIP3P water molecules [32]. The water box was extended 9 Å from the protein surface in all three dimensions. In order to neutralize the negative charge of the system, the sodium ions were added as required.

The solvated system was then energy-minimized using steepest-descent algorithm with 50000 steps to ensure the system has appropriate geometry. Equilibration of the solvated complex was done with NVT ensemble at 300 K for 400 ps and NPT ensemble at 1 bar for 400 ps.

The force constant of 1000 kJ/mol nm^2^ was used to position restrain all heavy atoms. The production simulations of 100 ns and 10 ns were performed for free EGFR models and EGFRs in complex with peptides under periodic boundary condition, respectively. The time step for all simulations was 2 fs.

The temperature and pressure were stabilized at 300 K and 1 bar applying V-rescale temperature [33] and Parrinello-Rahman pressure [34] coupling algorithms for both protein and solvent molecules. The time constant for temperature and pressure coupling was maintained on 0.1 and 2.0 ps, respectively. The cut-off value of 1 nm was applied to calculate the coulomb and van der Waals interactions. The long-range electrostatics were treated with (PME) Partial Mesh Ewald [35]. The long-range dispersion correction was applied for Energy and pressure. Parallel LINCS algorithm was applied to constrain all bonds in equilibration step [36].

### Analysis of MD simulations

MD simulation was visualized using VMD [37]. Root mean square deviation (RMSD), root mean square fluctuation (RMSF) and other parameters were obtained using GROMACS and the graphs visualization was performed using Grace-5.1.22/QTGrace v0.2.6 program.

### MM-PBSA calculation

In order to calculate the total binding free energies of the EGFR-peptide complexes, Molecular Mechanics–Poisson Boltzmann Surface Area (MM-PBSA) method was applied on the MD simulations [38]. G_mmpbsa tool was employed to calculate the free binding energies of the complexes [39]. The total binding free energy of each simulated EGFR-peptide complexes was calculated for 40 snapshots extracted every 0.25 ns from the whole production trajectories and then compared with EGFR-MIG6 complexes.

### Physicochemical properties

Physicochemical properties of the designed peptides were investigated using FAF-Drugs4 [40] web server. The physicochemical properties included MW, LogP calculated with XLOGP3 method, LogD at physiological pH (7.4), LogSw, HBD and HBA.

## Results

In the present study, molecular docking and dynamics simulation techniques in combination with MM-PBSA method have been employed to investigate the binding affinity of peptides in complex with both EGFRs. The results were compared with the crystal structures of WT-EGFR and EGFRL858R in complex with MIG6-pYpY (PDB IDs: 4ZJV and 4R3R respectively) and docked EGFR-MIG6YY complexes.

### Docking results

In order to investigate the binding modes and calculate the binding free energy, GRAMM-X [26] and Rosetta FlexPepDock server [27] were used to perform the docking between EGFRs and peptide library along with MIG6-YY. The protein-peptide docking results were compared in terms of interaction score and number of residues of the peptide-substrate binding site which were involved in interaction (Table 1).

**Table 1:** Computational modeling interaction scores and predicted interaction results of top scoring poses of docked peptides with WT-EGFR and EGFRL858R (PDB: 4ZJV, 4R3R).

Peptide name	Peptide sequences	Interaction scores (REU)		Number of residues*	
		WT-EGFR	EGFR L858R	WT-EGFR	EGFR L858R
MIG6-pYpY	THpYpYLLP	-	-	5	6
MIG6-YY	THYYLLP	-15.695	-16.941	8	7
5	STHHYYP	-16.599	-19.640	8	9
6	STHHYYL	-17.608	-19.131	7	7
10	HTHYYLP	-21.908	-20.212	10	8
26	KHTHYYD	-16.047	-19.982	6	8
27	KRTHYYD	-16.638	-20.093	8	6

REU: Rosetta Energy Unit is a standard energy unit used by FlexPepDock server to score complexes

* Indicates the number of amino acids in the EGFR peptide substrate binding site in interaction with the peptides.

Based on the structural investigations on the crystal structures of EGFR-MIG6pYpY complexes, MIG6-pYpY peptide covered 6 residues including Arg 841, Lys 875, Val 876, Pro 877, Lys 879 and Ala 920 in EGFRL858R complex and also covered the mentioned residues (except Arg 841) in WT-EGFR complex. (Table 1 and S1A1 and S1A2 Fig). The interaction scores for MIG6-YY peptide with WT-EGFR and EGFRL858R were -15.695 and -16.941 and the peptide covered the same residues along with Asp 142 and Ile 183 in EGFRL858R and the same residues along with Asp 145 and Ile 186 in WT-EGFR (Table 1 and S1B1 and S1B2 Fig).

**Fig 1:**
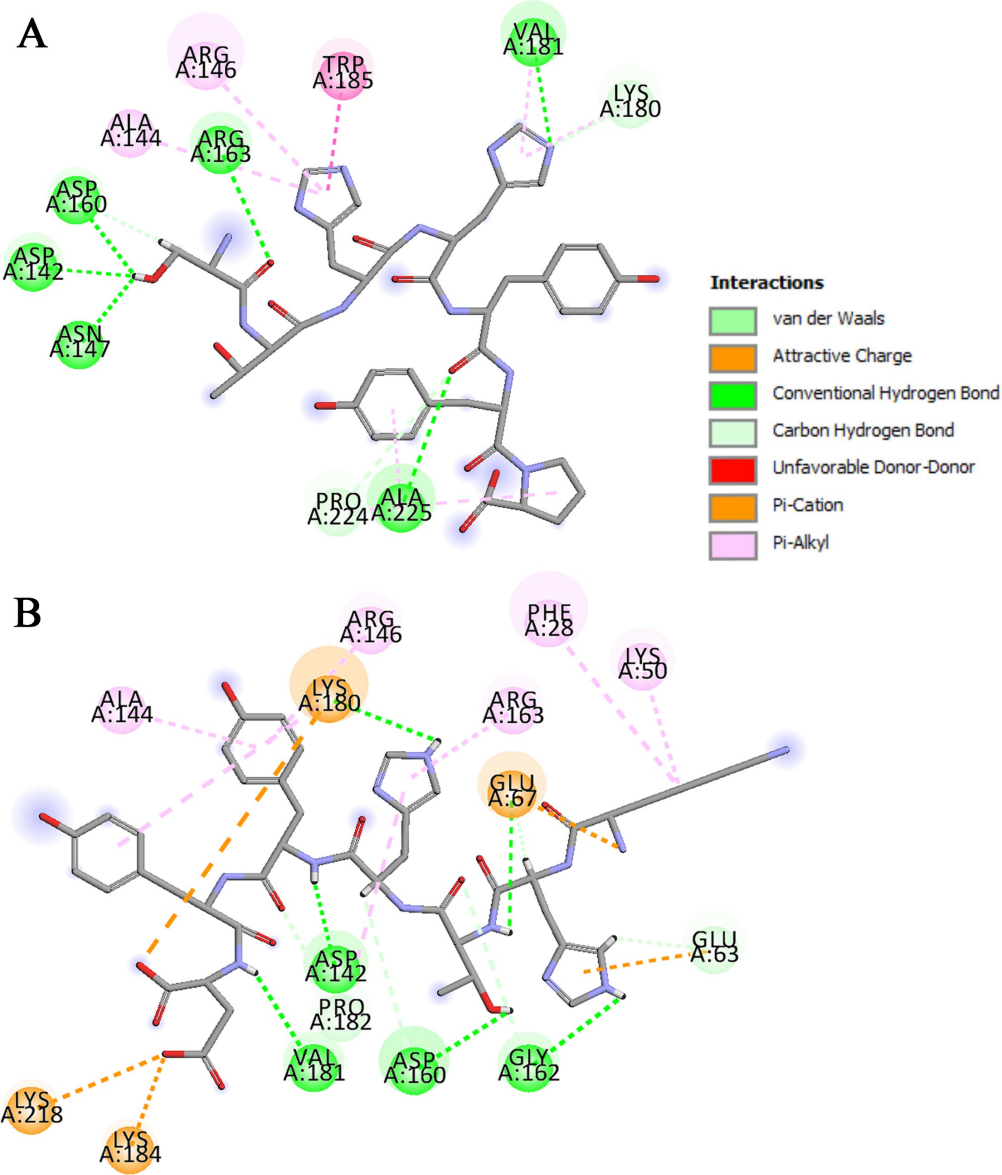
2D representation of predicted interaction between peptides and EGFR’s peptide-substrate binding site. Peptide 5 (A) and peptide 26 (B) in interaction with EGFRL858R. (A) peptide 5 covered 9 key residues of the peptide-substrate binding site, Asp 142, Arg 146, Asn 147, Asp 160, Arg 163, Lys 180, Val 181, Trp 185 and Ala 225. (B) Peptide 26 covered 8 key residues of EGFR kinase domain, Asp 142, Arg 146, Asp 160, Arg 163, Lys 180, Val 181, Pro 182, Lys 184.

**Fig 2:**
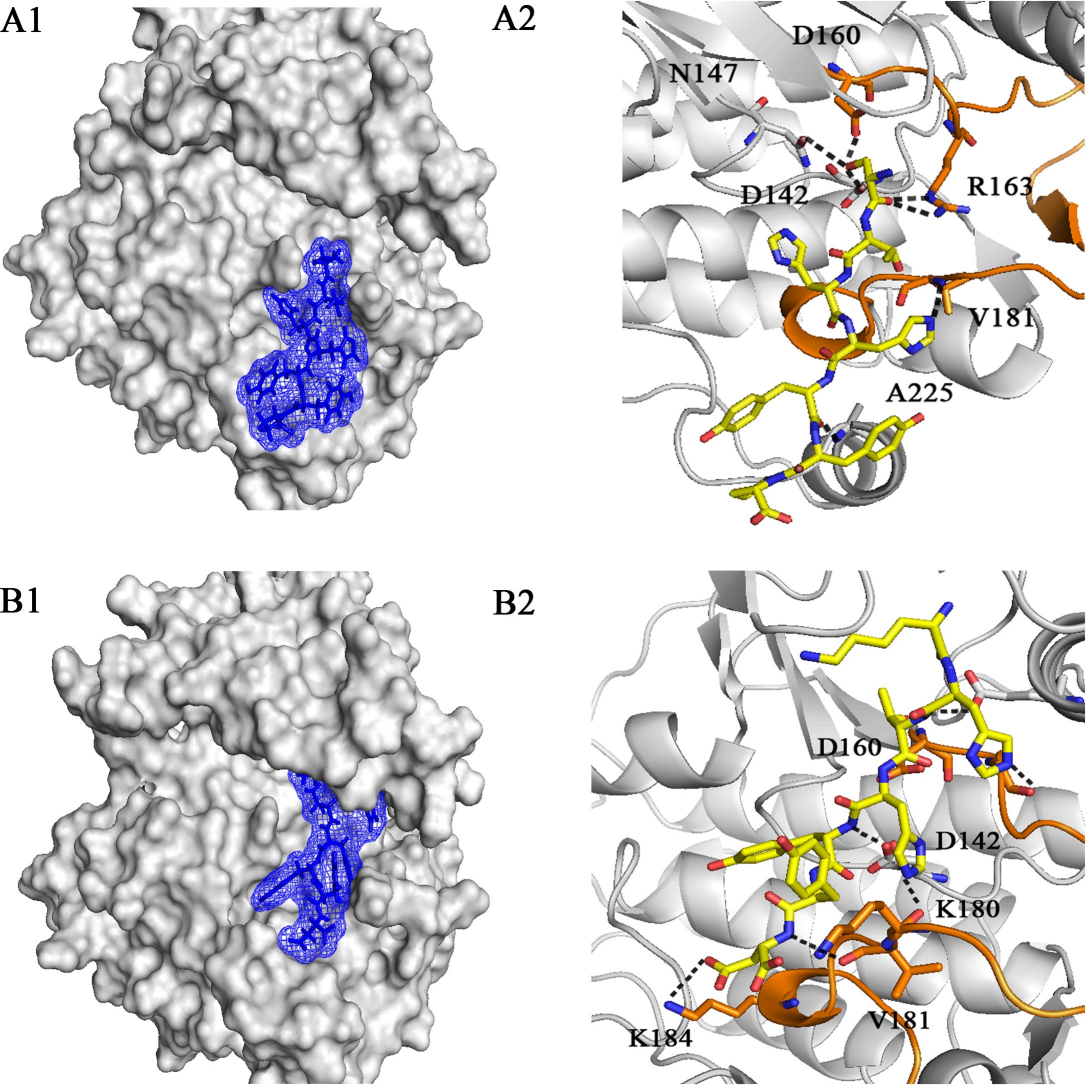
3D representation of peptides 5 and 26 in interaction with the peptide-substrate binding site of EGFRL858R. Overview and detailed view of interactions of peptide 5 (A1 and A2) and peptide 26 (B1 and B2) with EGFRL858R. (A2) Peptide 5 formed 7 hydrogen bonds with residues, Asp 142 to Ala 225 of the peptide-substrate binding site. (B2) peptide 26 formed 7 hydrogen bonds with residues, Asp 142 to Lys 184 of EGFR kinase domain. The peptides are shown in stick model. Black dotted lines indicate hydrogen bonds. The key residues of peptide-substrate binding site are illustrated in stick model.

In addition, the docking results showed that EGFRL858R had a higher binding affinity than WT-EGFR for peptides 5 (STHHYYP), 6 (STHHYYL), 26 (KHTHYYD) and 27 (KRTHYYD), while the binding affinity of both receptors for peptide 10 (HTHYYLP) was very close to each other. Among these peptides, Peptides 5 and 26 (compared to MIG6), showed higher interaction scores (-19.640 and -19.982) and covered the almost whole residues of peptide-substrate binding site in EGFRL858R, including Asp 142, Arg 146, Asn 147, Asp 160, Arg 163, Lys 180, Val 181, Pro 182, Lys 184, Trp 185, Ala 225 (Fig 1A and 1B). Then, they were considered to be investigated for the next studies (MD simulation and MM-PBSA). 2D interaction diagrams of these peptides in interaction with WT-EGFR are shown in S1F and S1G Fig. Fig 2 illustrates the 3D representation of peptides 5 and 26 in complex with EGFRL858R. As can be seen in Fig 2A2 and 2B2, peptide 5 formed seven hydrogen bonds with 6 key residues including Asp 142, Asn 147, Asp 160, Arg 163, Val 181 and Ala 225 and peptide 26 formed seven hydrogen bonds with 5 key residues including Asp 142, Asp 160, Lys 180, Val 181 and Lys 184 of peptide-substrate binding site of EGFRL858R. According to Figs 1 and 2, both peptides occupied the phosphoacceptor site (Asp 142) and the priming recognition pocket (Lys 184 and Ala 225).

Furthermore, peptides 6, 10 and 27 with interaction scores -19.131, -20.212, -20.093 and covered 7, 8 and 6 residues of EGFRL858R, respectively were also considered for the next studies (S1C1, S1D1 and S1E1 Fig). 2D interaction diagrams of these peptides in interaction with WT-EGFR are shown in S1C2, S1D2 and S1E2 Fig.

### Molecular dynamics simulations of EGFRTK complexes

In order to investigate the structural characteristics of EGFRL858R and WT-EGFR in free models and in complex with peptides, MD simulation has been done [41] and then MM-PBSA was employed to calculate the binding energy of complexes [41, 42]. Root Mean Square Deviation (RMSD) and Root Mean Square Fluctuation (RMSF) graphs were obtained for each MD simulation.

**Fig 3:**
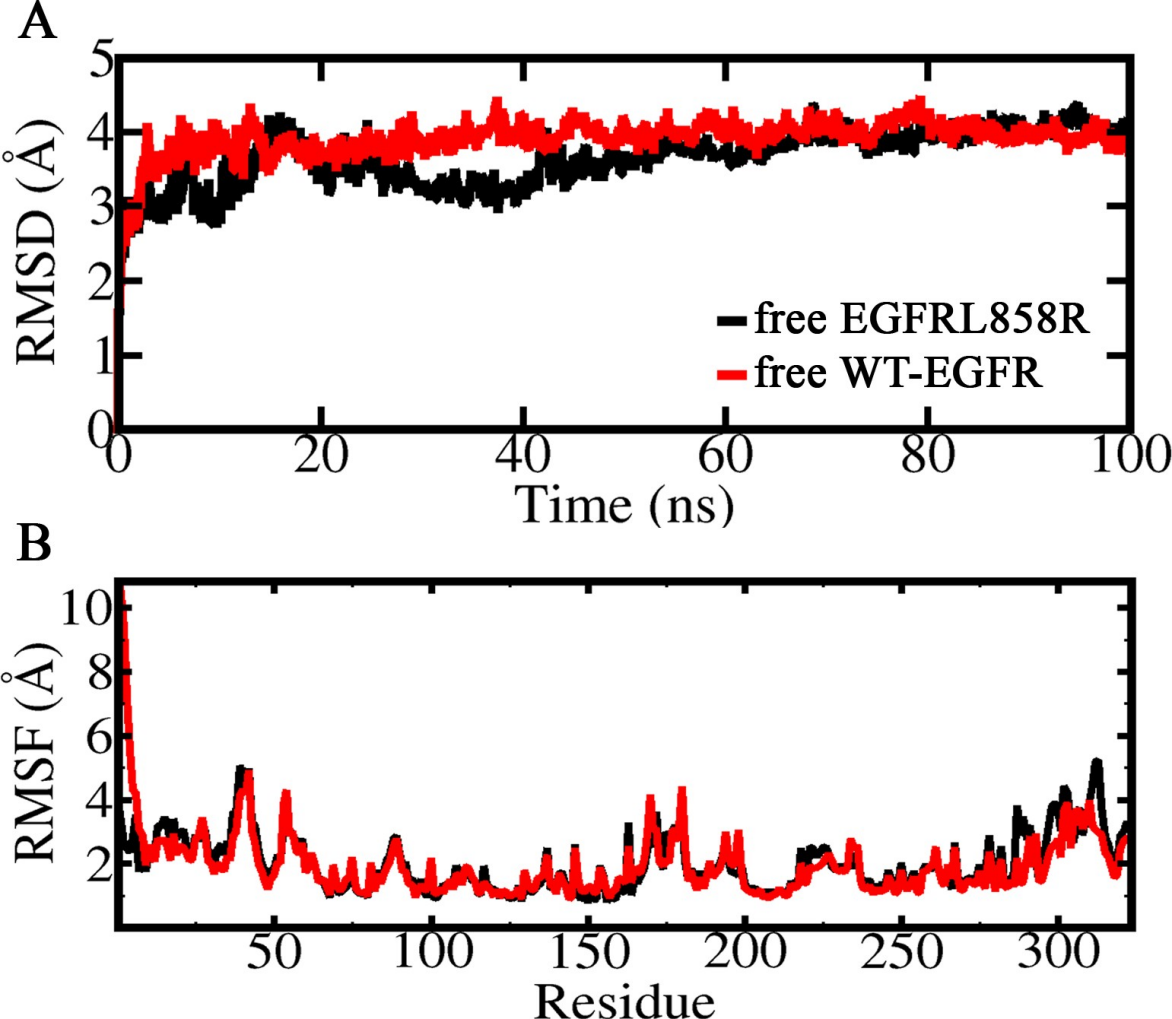
RMSD and RMSF of free EGFRs. (A) All atom RMSDs and (B) RMSFs of free EGFRL858R and WT-EGFR during 100 ns simulation.

The RMSD and RMSF graphs for both free receptors (WT-EGFR and EGFRL858R) are depicted in Fig 3. The all-atom RMSD values for free models of both EGFRs relative to initial structures indicated that EGFRL858R and WT-EGFR are stabilized around 4 Å after approximately 20 ns of the simulation (Fig 3A). The RMSF graphs of free EGFR models revealed that there is no considerable difference in flexibility of both receptors, except in activation loop (residues 160–190) and residues 300–320 (Fig 3B). The RMSD graph of each EGFRs in complex with peptides (MIG6, 5, 6, 10, 26 and 27) indicated that EGFRL858R complexes were more stable than WT-EGFR during the simulation. Also WT-EGFR in complex with peptides 6 and 27 showed higher RMSD values. (S2A and S2B Fig).

Also, the RMSF graphs of EGFR-peptide complexes showed that the most flexible regions of WT-EGFR and EGFRL858R were related to the loop shape region located in N-lobe (residues 37–48), activation loop (residues 160–190) and residues 300–320 (S3A and S3B Fig).

**Fig 4:**
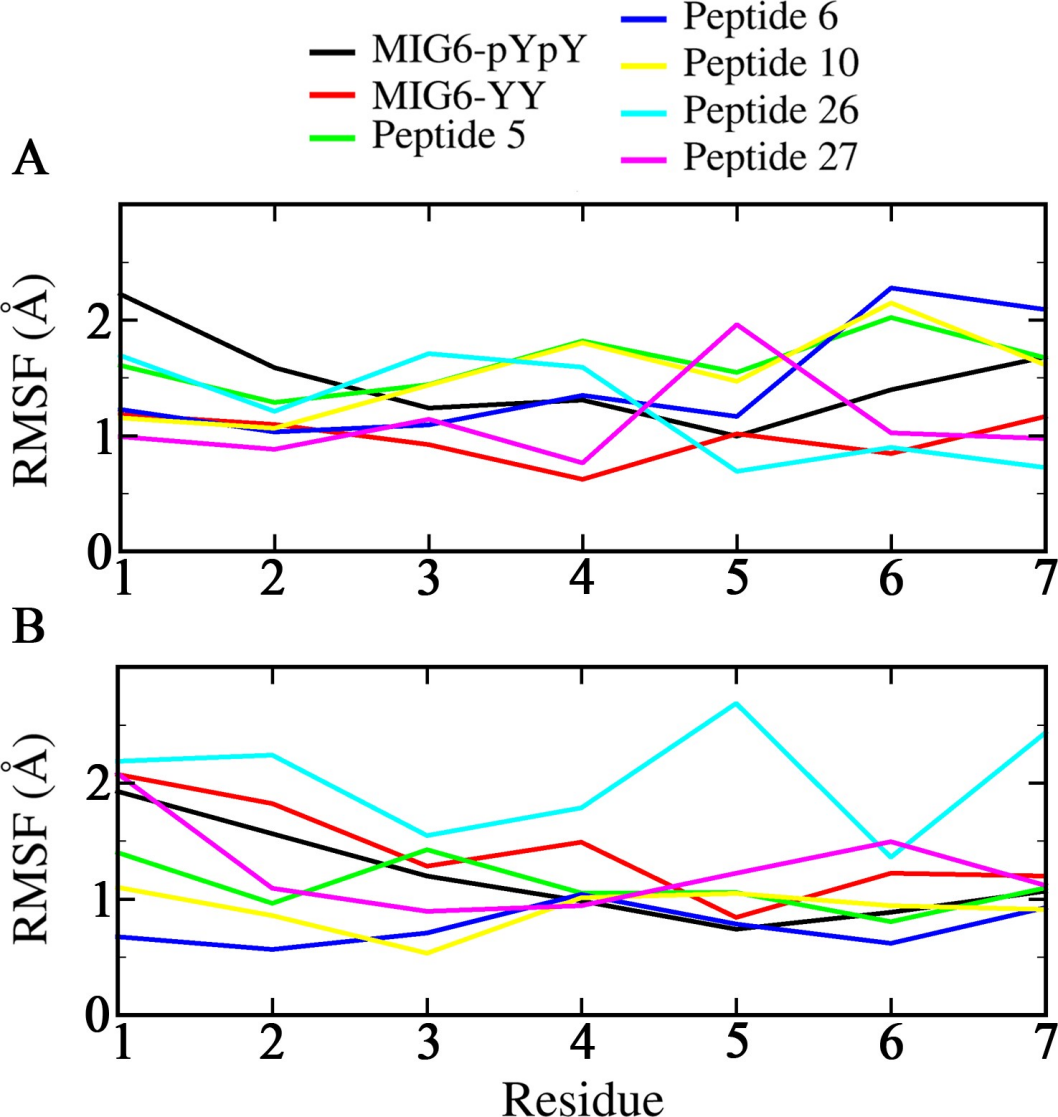
RMSF graph of all peptides in interaction with both EGFRs. Peptides MIG6-pYpY, MIG6-YY, 5, 6, 10, 26 and 27 in interaction with (A) EGFRL858R and (B) WT-EGFR.

Furthermore, the RMSF values of the peptides were depicted in Fig 4. As can be seen in Fig 4A, there was no the remarkable difference between the flexibility of the peptides in complex with EGFRL858R. While the difference in flexibility of the peptides in complex with WT-EGFR was observed which was related to peptide 26 with the highest fluctuation in Tyr 5 and Asp 7 (Fig 4B).

### Calculation of binding energy and per-residue energy contribution

In order to calculate the total binding free energy and per-residue energy contribution, we applied the G_MMPBSA package [39]. The estimated binding energies for the WT-EGFR and EGFRL858R in complex with the peptides MIG6, 5, 6, 10, 26 and 27 are shown in Table 2.

**Table 2:** Total binding free energies of WT-EGFR and EGFRL858R in complex to MIG6-YY, Peptides 5, 6, 10, 26 and 27 obtained by MM-PBSA method.

Total binding free energy (Kcal/mol)			
Peptide number	Peptide sequences	WT-EGFR	EGFRL858R
MIG6-YY	THYYLLP	-16.397	-18.706
Peptide5	STHHYYP	-4.497	-23.923
Peptide6	STHHYYL	-30.592	-9.205
Peptide10	HTHYYLP	-15.675	-44.791
Peptide26	KHTHYYD	-8.544	-25.098
Peptide27	KRTHYYD	-45.258	-46.426

**Fig 5:**
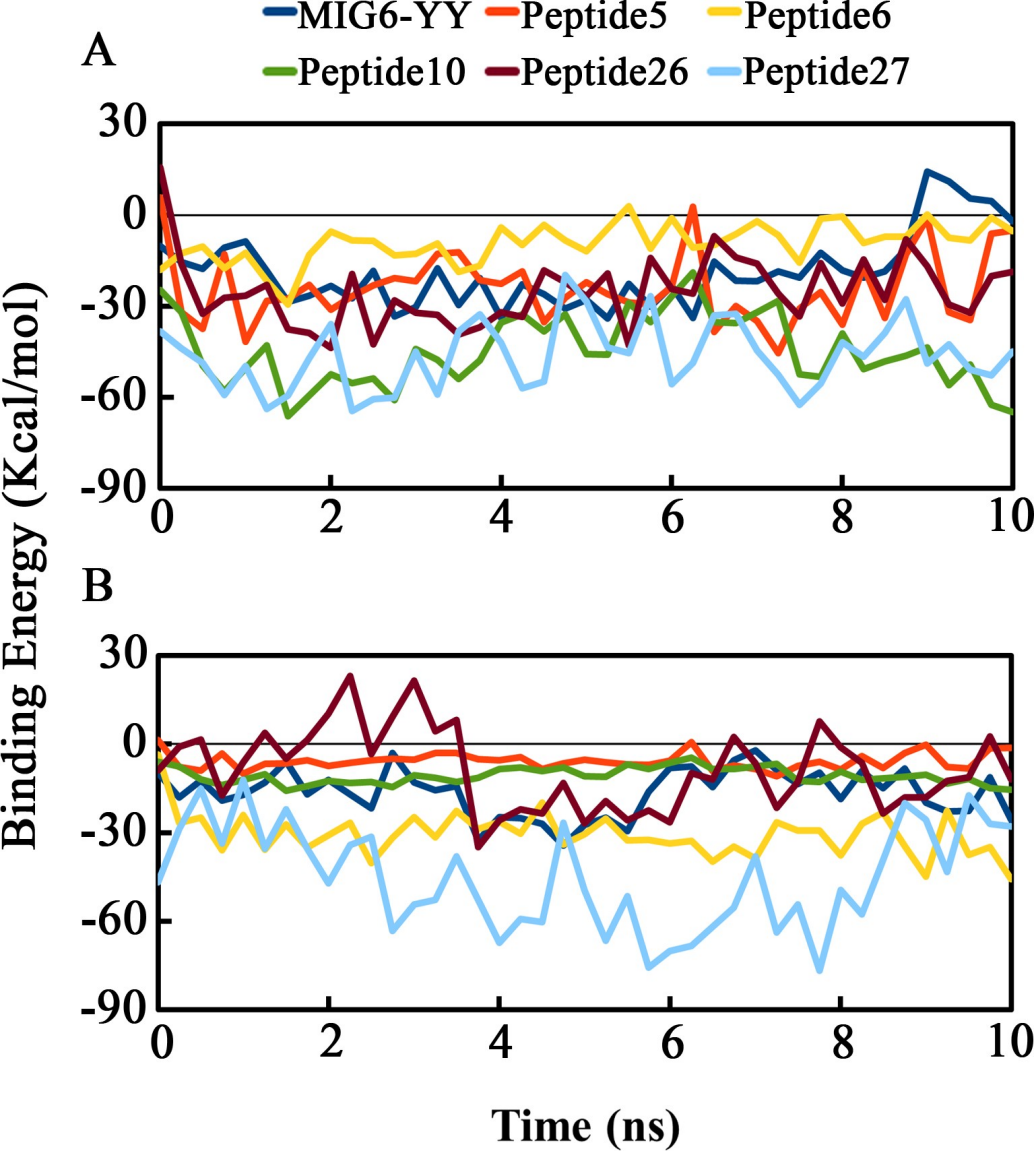
Total binding free energies of both EGFRs in complex with peptides during 10 ns using MM-PBSA method. (A) EGFRL858R and (B) WT-EGFR in complex with MIG6-YY, Peptides 5, 6, 10, 26 and 27. (A) Peptides 5, 10, 26 and 27 have the highest binding affinities to EGFRL858R (-23.923, -44.791, -25.098 and -46.426 Kcal/mol, respectively). (B) peptides 6 and 27 have the highest binding affinities to WT-EGFR (-30.592 and -45.258 Kcal/mol, respectively). Peptide 27 with binding energies (-46.426 and -45.258 Kcal/mol) has similar binding affinities to EGFRL858R and WT-EGFR, respectively.

Based on the MM-PBSA results, despite peptide 27 is the most potent EGFR tyrosine kinase inhibitor but it is not able to inhibit EGFRL858R selectively, while peptides 5, 10 and 26 showed higher binding affinity to EGFRL858R rather than WT-EGFR. On the other hand, peptide 6 has higher binding affinity to WT-EGFR rather than EGFRL858R. Fig 5 demonstrates the total binding energies of both receptors in complex with all the peptides during the simulation.

Peptides 5 and 26 with binding affinities -23.923 Kcal/mol and -25.098 Kcal/mol, respectively had higher binding affinity than MIG6 (-18.706 Kcal/mol) to EGFRL858R that was in agreement with the docking results. In addition, peptide 10 with binding affinity -44.791 Kcal/mol showed higher affinity than MIG6 and the rest of peptides to EGFRL858R.

**Fig 6:**
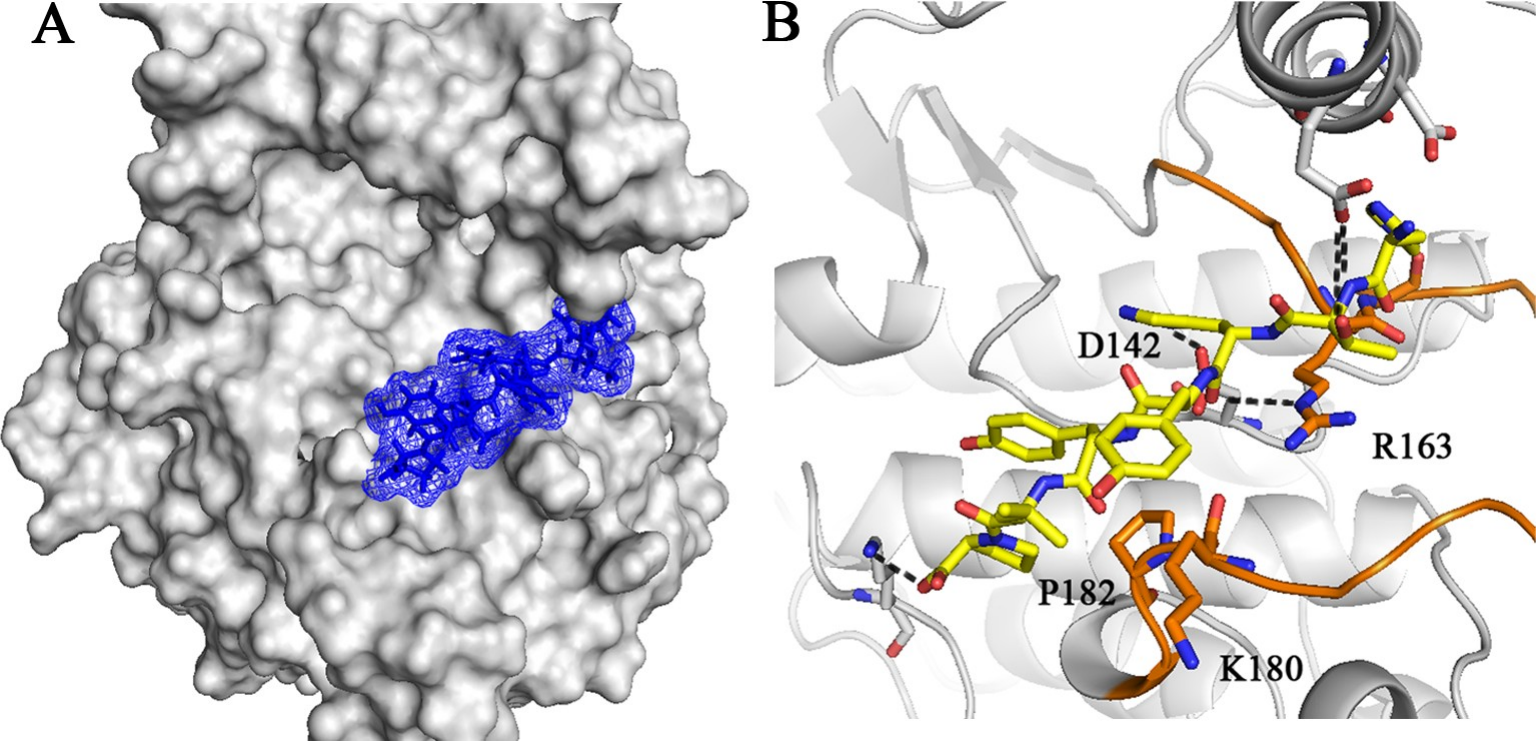
3D representation of peptide 10 in interaction with EGFRL858R. Overview (A) and detailed view (B) of interactions of peptide 10 with the peptide-substrate binding site of EGFRL858R. (B) peptide 10 formed five hydrogen bonds with 4 key residues of EGFR kinase domain, Asp 142, Arg 163, Lys 180 and Pro 182 and occupied the phosphoacceptor site by a hydrogen bond between His 3 and Asp 142. Peptide 10 is shown in stick model. Black dotted lines indicate hydrogen bonds. The key residues of peptide-substrate binding site are illustrated in stick model.

3D representation of peptide 10 in complex with EGFRL858R is illustrated in Fig 6. As can be seen in Fig 6B, five hydrogen bonds are formed between peptide 10 and peptide-substrate binding site of EGFRL858R including, Asp 142, Arg 163, Lys 180 and Pro 182. 2D diagram of this peptide in interaction with peptide-substrate binding site of EGFRL858R was depicted in S1D1 Fig. Peptide 10 covered 8 key residues of EGFR kinase domain including, Asp 142, Arg 146, Asn 147, Arg 163, Lys 180, Pro 182, Lys 184 and Trp 185. According to Fig 6B and S1D1 Fig, peptide 10 occupied the phosphoacceptor site through hydrogen bond between His 3 and Asp 142, the catalytic base.

**Fig 7:**
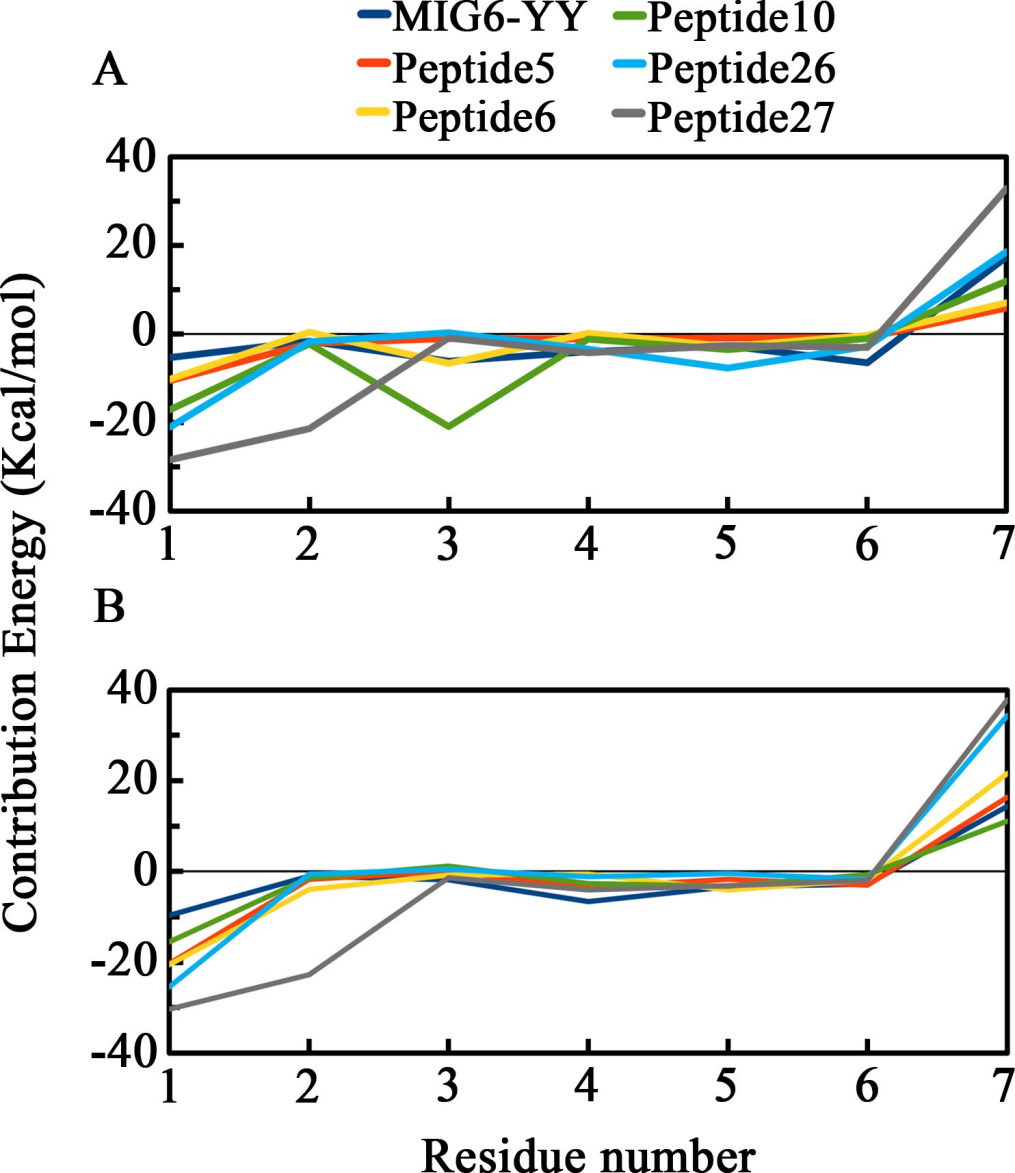
**Per-residue energy contribution of peptides MIG6-YY, 5, 6, 10, 26 and 27 in interaction with EGFRL858R (A) and WT-EGFR (B).** Peptide 10 (His 1 and His 3), peptide 5 (Ser 1) and peptide 26 (Lys 1 and Tyr 5) have higher contribution in interaction with the peptide-substrate binding site of EGFRL858R (A). peptide 6 (Ser 1) has higher contribution in interaction with the peptide-substrate binding site of WT-EGFR (B). Peptide 27 has higher contribution in interaction with both receptors through Lys 1 and Arg 2.

Binding energy decomposition of peptides have been calculated during the simulations to determine the contribution of each residue of the peptides to the overall binding energy. To this purpose, per-residue energy contribution of the peptides MIG6-YY (THYYLLP), 5 (STHHYYP), 6 (STHHYYL), 10 (HTHYYLP), 26 (KHTHYYD) and 27 (KRTHYYD) in complex with WT-EGFR and EGFRL858R were calculated using G_MMPBSA (Fig 7). According to the results, His 1 and His 3 of peptide 10, Ser 1 of peptide 5, Lys 1 and Tyr 5 of peptide 26 have contributed more in binding to the peptide-substrate binding site of EGFRL858R (Fig 7A). While Ser 1 of peptide 6 has contributed more in binding to the peptide-substrate binding site of WT-EGFR and the residues of Lys1 and Arg 2 of peptide 27 interacted tightly to both receptors (Fig 7B).

### Physicochemical properties

In order to calculate the physicochemical properties of the designed peptides, FAF-Drugs4 [40] web server was used. Based on calculated physicochemical properties in S3 Table, peptides 5, 6 and 10 indicated the higher LogP and LogD values compared to peptides 26 and 27 and peptides 26 and 27 had the most solubility.

## Discussion

MIG6 molecule (segments 1+2) acts as an activated EGFR tyrosine kinase inhibitor and inhibits it by blocking the asymmetric dimer association through its segment 1 and occupying the peptide-substrate binding site via segment 2. The Y394-Y395 phosphorylation site in segment 2 plays the critical role to detect and bind tightly to the active EGF receptor and inhibit it. An interesting property of MIG6 is its ability in inhibiting constitutively active receptor, EGFRL858R, that is much more potent than its ability in inhibiting the basal activity of the WT-EGFR [3, 6]. The rate of dissociation for the EGFR-Mig6pYpY complex is estimated about 45 min, which is sufficiently longer and slower than the time required (5–10 minutes) for internalization of activated EGFR in cell [3].

Peptide-substrate binding site is a shallow cleft with solvent exposed residues, Asp 837, Arg 841, Asn 842, Asp 855, Lys 875, Val 876, Pro 877, Ile 878, Lys 879, Trp 880, Ser 885 and Ala 920 which is our target in this *in silico* study to design inhibitory peptides in order to inhibit EGFRL858R.

To this purpose, we first provided a peptide library comprising of designed peptides considering the MIG6 segment 2 structure which its YY motif is maintained in their sequences, then performed molecular docking and molecular dynamic simulation studies on both WT-EGFR and EGFRL858R. The results of this study were compared with crystal structure of EGFR-MIG6 pYpY and docked EGFR-MIG6YY complexes.

Docking techniques were applied to perform local docking of the designed peptides and MIG6-YY with WT-EGFR and EGFRL858R. The docking results suggest that peptides 5 (STHHYYP) and 26 (KHTHYYD) could be considered as selective inhibitors for EGFRL858R, since these peptides had higher binding affinities (-19.640 and -19.982) to EGFRL858R rather than WT-EGFR (Table 1). In addition, they were able to cover more residues from peptide-substrate binding site, particularly the key residues including the catalytic base, Asp 142 (phosphor-acceptor site) and Lys 184 and Ala 225 (priming recognition pocket) (Fig 1A and 1B).

Peptide 10 (HTHYYLP) showed approximately similar binding affinities to both receptors (-20.212 and -21.908) and peptides 6 (STHHYYL) and 27 (KRTHYYD) covered nearly similar residues of peptide-substrate binding site as MIG6. These peptides were also considered to be investigated for the next studies because of their high binding affinities (-20.212, -19.131 and -20.093 respectively), in complex with EGFRL858R. (Table 1).

Molecular dynamics simulation was performed to investigate the stability and conformational changes of free EGFR models and EGFR in complex forms.

RMSD and RMSF plots for free EGFRs showed that there is no significant dynamically differences between both receptors, and they are stable during the simulation with RMSD value of around 4 Å (Fig 3A and 3B).

Comparison of RMSD plots of free EGFRs and EGFR-peptide complexes showed that both receptors (particularly EGFRL858R) in complex with peptides are more stable than free EGFRs during the simulation (S4A and S4B Fig).

Comparison of RMSF plots for free EGFRs and EGFR-peptide complexes revealed the difference in local flexibility which is related to the peptide-substrate binding site (residues 180–185). The flexibility of this region is higher in free EGFRs than EGFR complexes (S5A and S5B Fig).

The RMSF plots of the peptides in complex with both receptors indicated that all peptides (except peptide 26) showed more stability in complex with WT-EGFR than in complex with EGFRL858R and the lowest fluctuation is observed in their 4 last residues. While there is no significant difference in flexibility of the peptides in complex with mutant EGFR (Fig 4A and 4B).

The binding free energy of EGFR-peptide complexes was calculated using MM-PBSA.

The results indicated that peptides 5, 10 and 26, have significant binding affinity to EGFRL858R than WT-EGFR, compared to MIG6-YY (Table 2). These data, in agreement with the docking results, are suggesting that these peptides could be more potent inhibitory peptides than MIG6-YY to inhibit EGFRL858R, particularly peptides 5 and 10. Peptide 5 was able to cover the most residues of peptide-substrate binding site and peptide 10 had much higher binding affinity to EGFRL858R, compared to MIG6. (S2 Table).

In addition, per-residue energy contributions of peptides MIG6-YY, 5, 6, 10, 26 and 27 in complex with both receptors were calculated. According to the results, Ser 1 of peptide 5, His 1 and His 3 of peptide 10 and Lys 1 of peptide 26 strongly interacted with the peptide-substrate binding site of EGFRL858R (Fig 7A). Also, peptide 10 with binding energy -44.791 Kcal/mol interacted strongly to the peptide-substrate binding site of EGFRL858R through its residues His 1 and His 3 (Table 2 and Fig 7).

The *in silico* prediction of the physicochemical properties of the designed peptides suggested that peptides 5, 6 and 10 are suitable peptides to inhibit EGFRL858R compared to MIG6s (S3 Table).

Based on the present study, it can be concluded that peptides 5 (STHHYYP) and 10 (HTHYYLP) as potential inhibitory peptides could be considered as mutant-selective inhibitors and are able to inhibit EGFRL858R more potently than MIG6-YY. Peptide 5 occupied both pockets (phosphoacceptor and priming recognition pockets) of peptide-substrate binding site (Figs 1A and 2A2) and peptide 10 with highest binding energy occupied the phosphoracceptor site (Fig 6B).

Given that, both subunits of EGFRL858R asymmetric dimer are presumably active and contribute to EGFR signaling, MIG6 molecule is not expressed sufficiently to bind both active kinases [3], therefore it is predicted that peptides 5 and 10 with higher affinity than MIG6-YY peptide could potently inhibit EGFRL858R.

## Conclusion

The present *in silico* study is the first step towards designing inhibitory peptides according to the MIG6 peptide structure. Several advantages of peptides including ease of synthesis and modifications, low toxicity and the high target specificity and selectivity, make them desirable candidate for cancer treatment. Additionally, with respect to the disadvantages of TKIs, the inhibitory peptides are more suitable alternatives to small molecule inhibitors to cover the peptide-substrate binding site, because of their larger molecular size.

The findings of this study suggest that peptides 5 and 10 as qualified peptides could be considered as mutant-selective inhibitors and are able to inhibit EGFRL858R more potently than MIG6-YY. Providing experimental investigations of the desired synthesized peptides in combination with our computational studies will be needed to confirm and complete this study.

It is recommended to compare our computational results with MIG6-pYpY peptide *in vitro*. Also, it would be interesting to phosphorylate our inhibitory peptides 5 and 10 on their YY motif and evaluate the effectiveness of their inhibitory potential on EGFRs compared to MIG6-pYpY.

The data presented in this study could be helpful in the designing of novel inhibitory peptides to potently inhibit other mutant EGFR variants which do not respond to available EGFR TKIs.

## Supporting information

S1 Fig 2D diagrams of predicted interactions between EGFRs peptide-substrate binding site and selected peptides. The crystal structure of EGFRL858R-MIG6 pYpY complex (A1), the docked peptides MIG6-YY (B1), peptide 6 (C1), peptide 10 (D1) and peptide 27 (E1) with EGFRL858R. The crystal structure of WT EGFR-MIG6 pYpY complex (A2), the docked peptides MIG6-YY (B2), peptide 6 (C2), peptide 10 (D2) peptide 27 (E2), peptide 5 (F) and peptide 26 (G) with WT-EGFR. (A1) MIG6-pYpY in complex with the crystal structure of EGFRL858R covered only 6 residues of 12 key residues of peptide-substrate binding site, Arg 841, Lys 875, Val 876, Pro 877, Lys 879, Ala 920 and occupied the priming recognition pocket (Lys 879 and Ala 920). Docked peptides MIG6-YY, 6, 10 and 27 (B1, C1, D1 and E1) with EGFRL858R covered 7, 7, 8 and 6 residues of the binding site, respectively and all of them occupied both pockets, phosphoacceptor site (Asp 142) and priming recognition pocket (Lys 184 and Ala 225) except peptide 10) occupied only phosphoacceptor site(. (A2) MIG6-pYpY in complex with the crystal structure of WT-EGFR covered only 5 residues of 12 key residues of peptide-substrate binding site, Lys 875, Val 876, Pro 877, Lys 879, Ala 920 and occupied the priming recognition pocket (Lys 875 and Ala 920). Docked peptides MIG6-YY, 6, 10 and 27 (B2, C2, D2 and E2) with WT-EGFR covered 8, 7, 10 and 8 residues of the binding site, respectively. Docked peptides 5 and 26 (F and G) with WT-EGFR covered 8 and 6 residues of binding site, respectively.
(PDF)

S2 Fig RMSDs of EGFRL858R and WT-EGFR in complex with the peptides. All-atom RMSDs of EGFRL858R (A) and WT-EGFR (B) in complex with peptides MIG6-pYpY, MIG6-YY, 5, 6, 10, 26 and 27. EGFRL858R complexes were more stable than WT-EGFR during the simulation.
(JPG)

S3 Fig RMSF of EGFRL858R (A) and WT-EGFR (B) in complex with peptides MIG6-pYpY, MIG6-YY, 5, 6, 10, 26 and 27. The most flexible regions of WT-EGFR and EGFRL858R were related to the loop shape region located in N-lobe (residues 37–48), activation loop (residues 160–190) and residues 300–320.
(JPG)

S4 Fig Comparison of RMSD of free EGFRs and EGFR-peptide complexes. All-atom RMSD of EGFRL858R in free form, and in complex with peptides MIG6-pYpY, MIG6-YY, 5, 6, 10, 26, 27 (A) and RMSD of WT-EGFR in free form, and in complex with MIG6-pYpY, MIG6-YY, 5, 6, 10, 26, 27 (B). EGFR complexes (particularly EGFRL858R) are more stable than free EGFRs during the simulation.
(JPG)

S5 Fig Comparison of RMSF plots of free EGFRs and EGFR-peptide complexes. RMSF plot of EGFRL858R in free form and in complex with peptides MIG6-pYpY, MIG6-YY, 5, 6, 10, 26, 27 (A) and RMSF plots of WT-EGFR in free form and in complex with MIG6-pYpY, MIG6-YY, 5, 6, 10, 26, 27 (B). The flexibility of the peptide-substrate binding site comprising residues 180 to 185 is higher in free EGFRs than EGFR-peptide complexes.
(JPG)

S1 Table Binding scores of top scoring poses with WT-EGFR and EGFRL858R (PDB: 4ZJV, 4R3R). (PDF)

S2 Table Comparison of WT-EGFR and EGFRL858R total binding energy calculated by Rosetta FlexPepDock and MM-PBSA. (PDF)

S3 Table Physicochemical properties of peptides MIG6-pYpY, MIG6-YY, 5, 6, 10, 26 and 27. (PDF)
