# Supplementary figures and images for: Structure-based inhibitory peptide design targeting peptide-substrate binding site in EGFR tyrosine kinase
Source: PLoS One. 2019 May 22;14(5):e0217031. doi: 10.1371/journal.pone.0217031 (PMC6530890; doi:10.1371/journal.pone.0217031)

A1

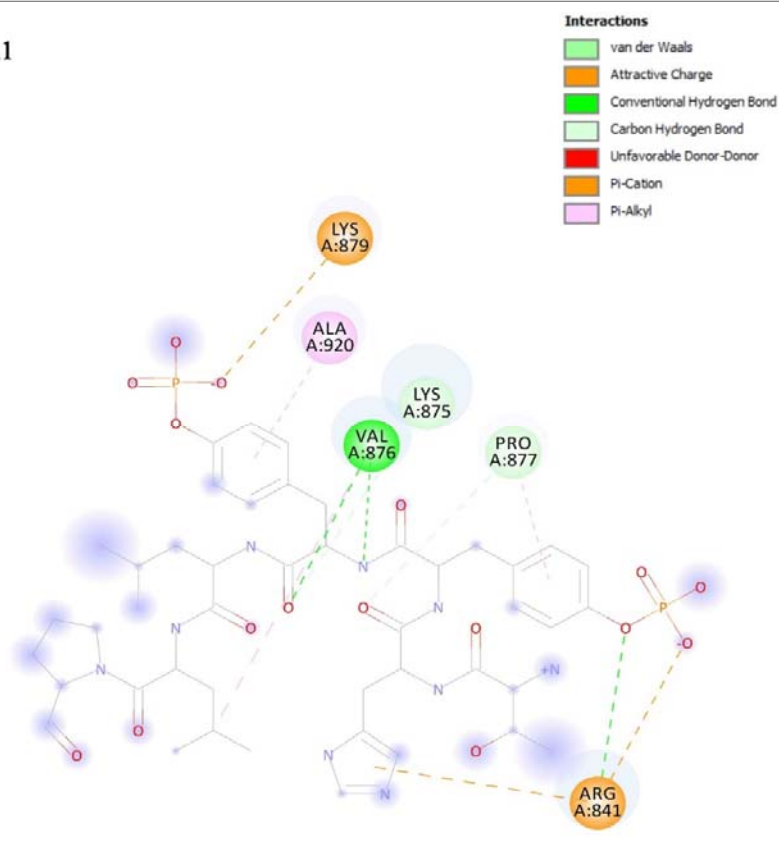

A2

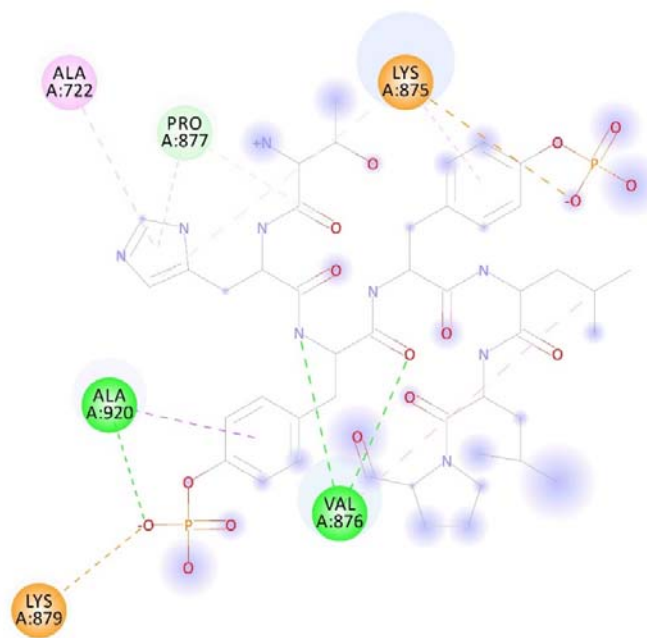

B1

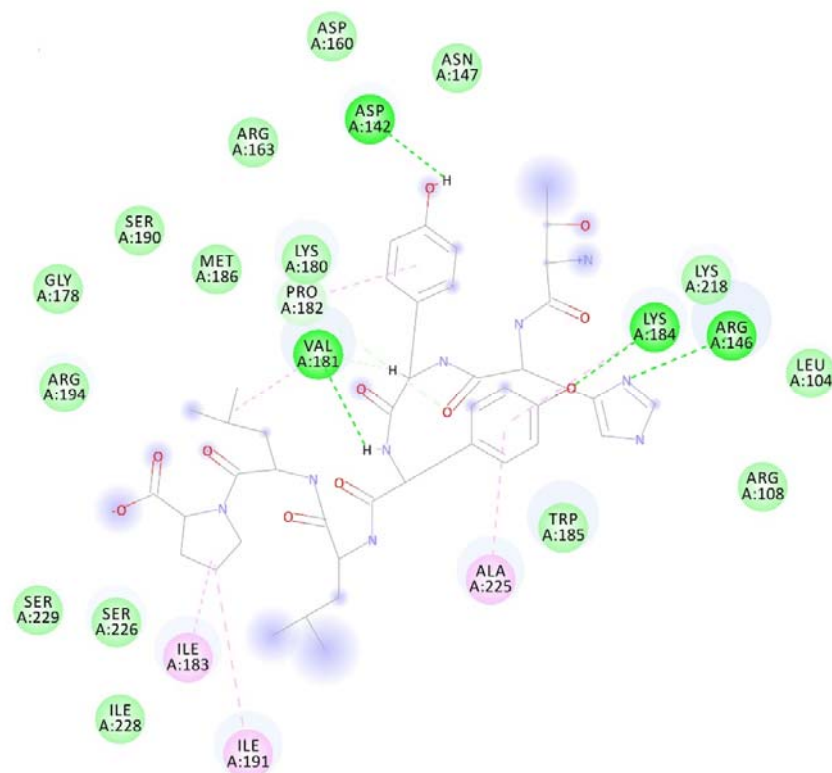

B2

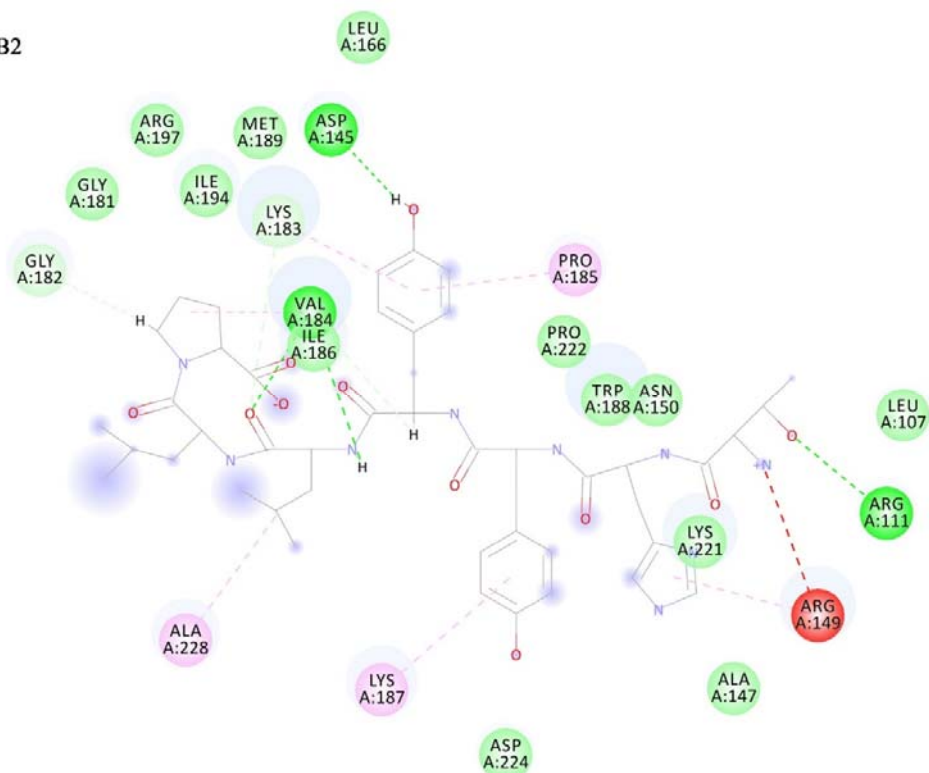

C1

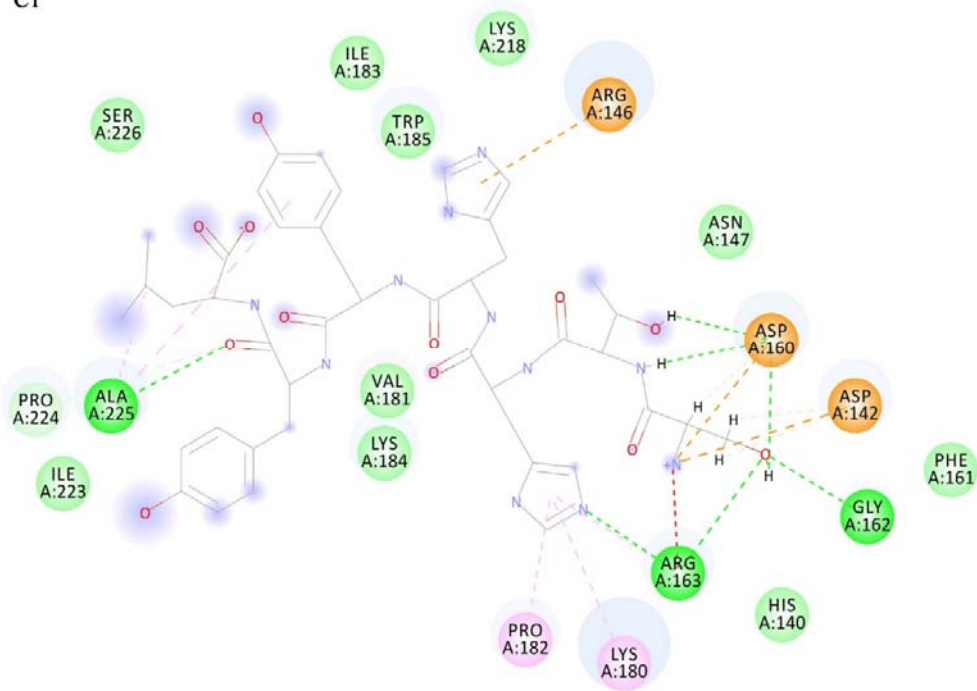

C2

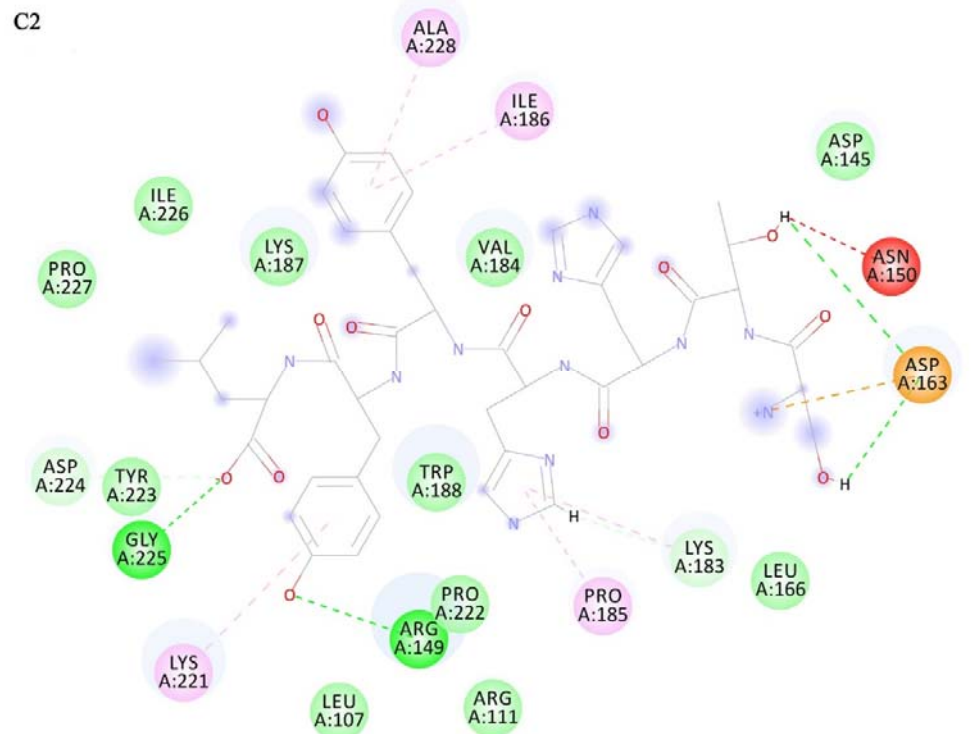

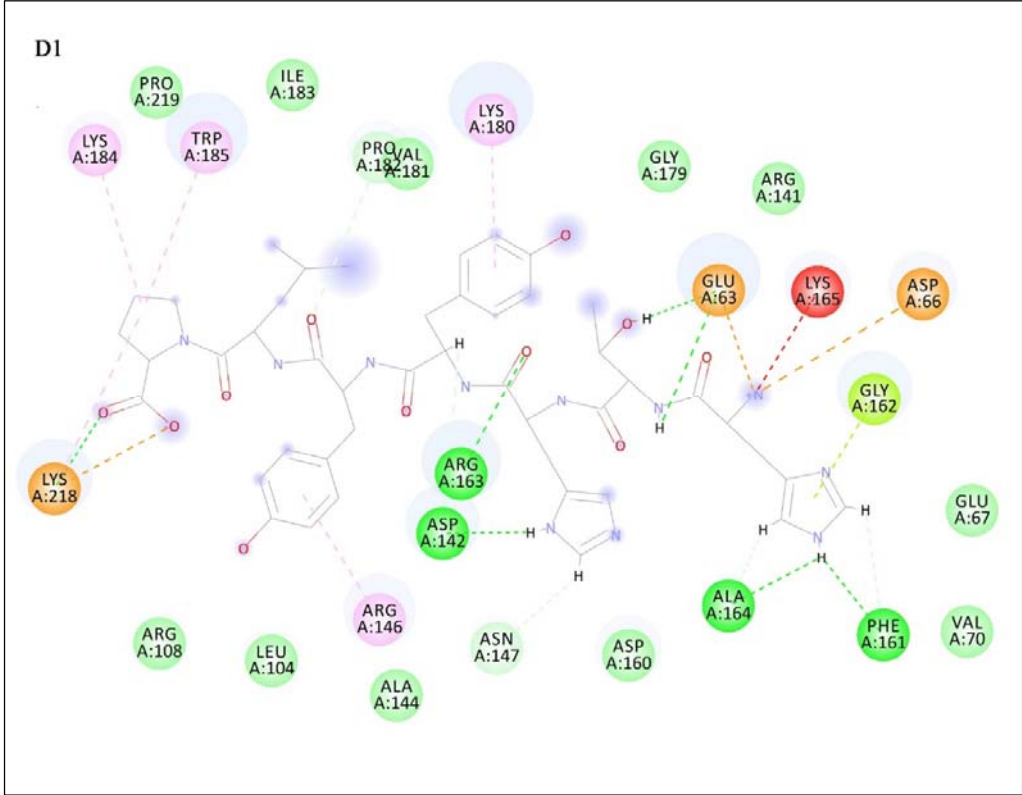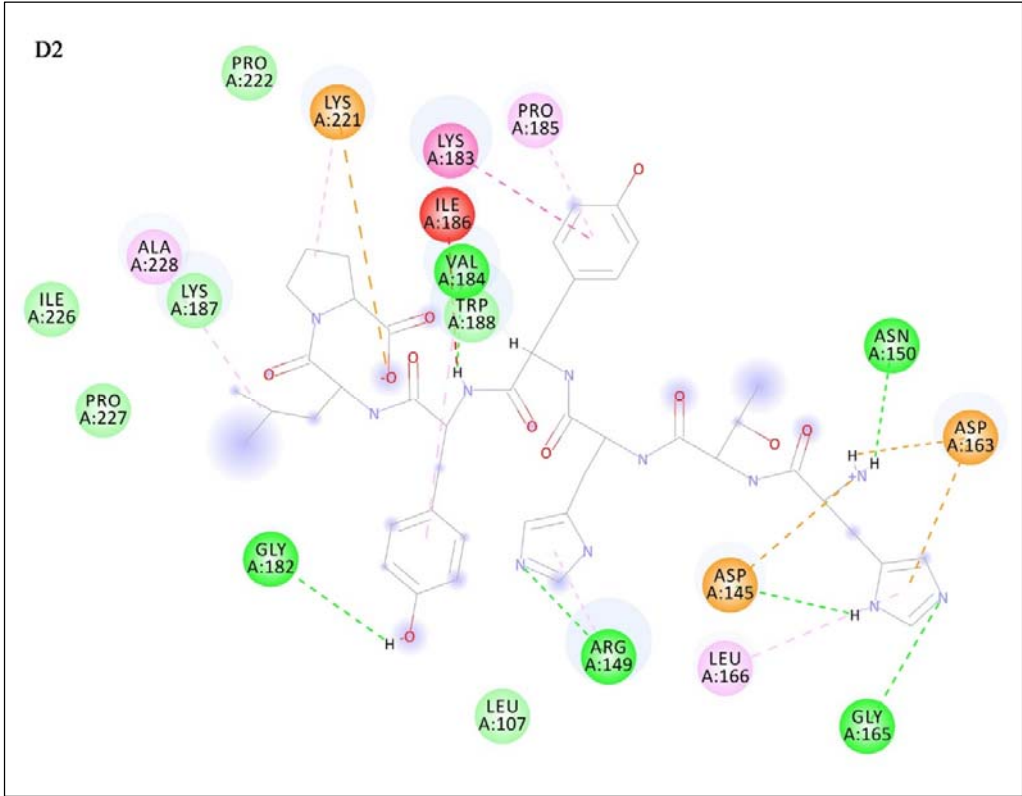

E1

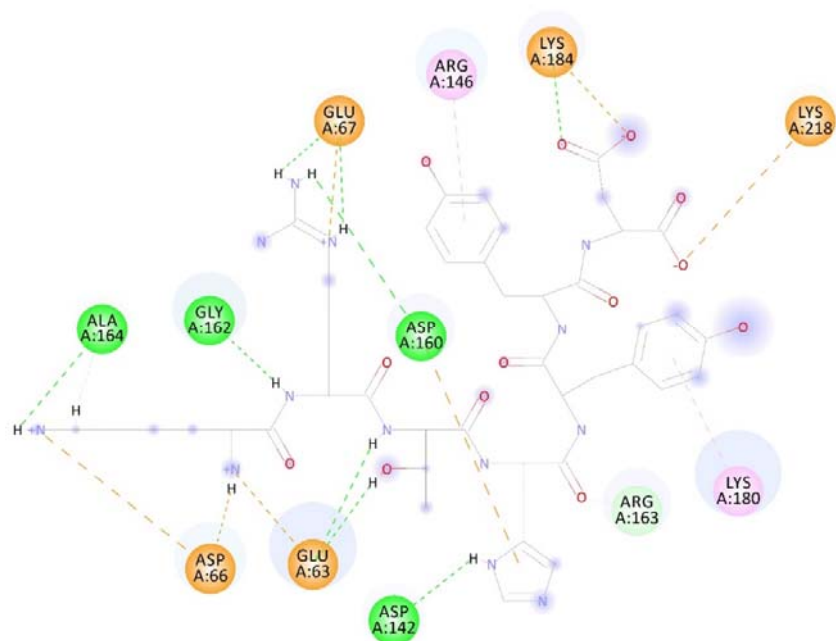

E2

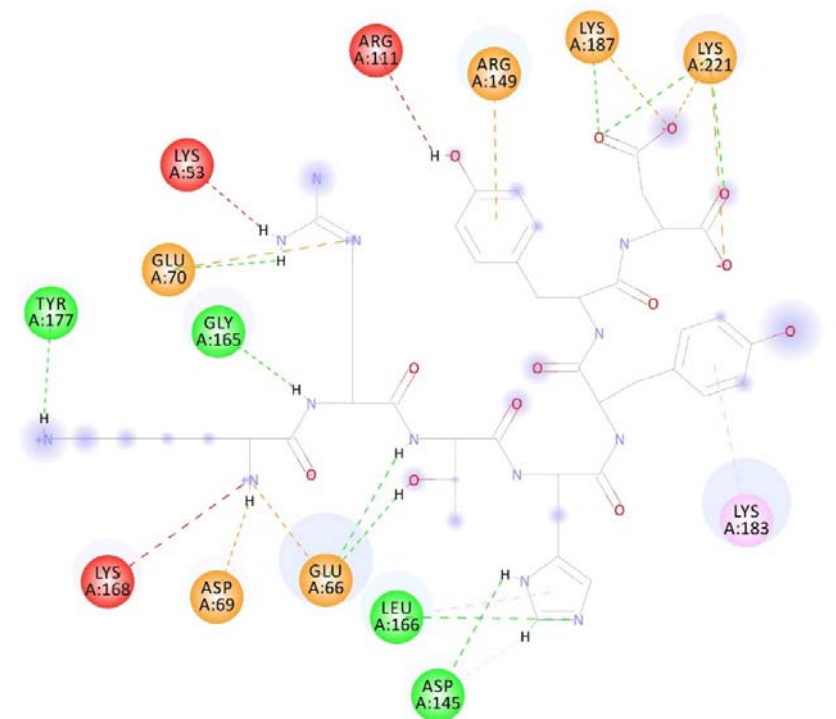

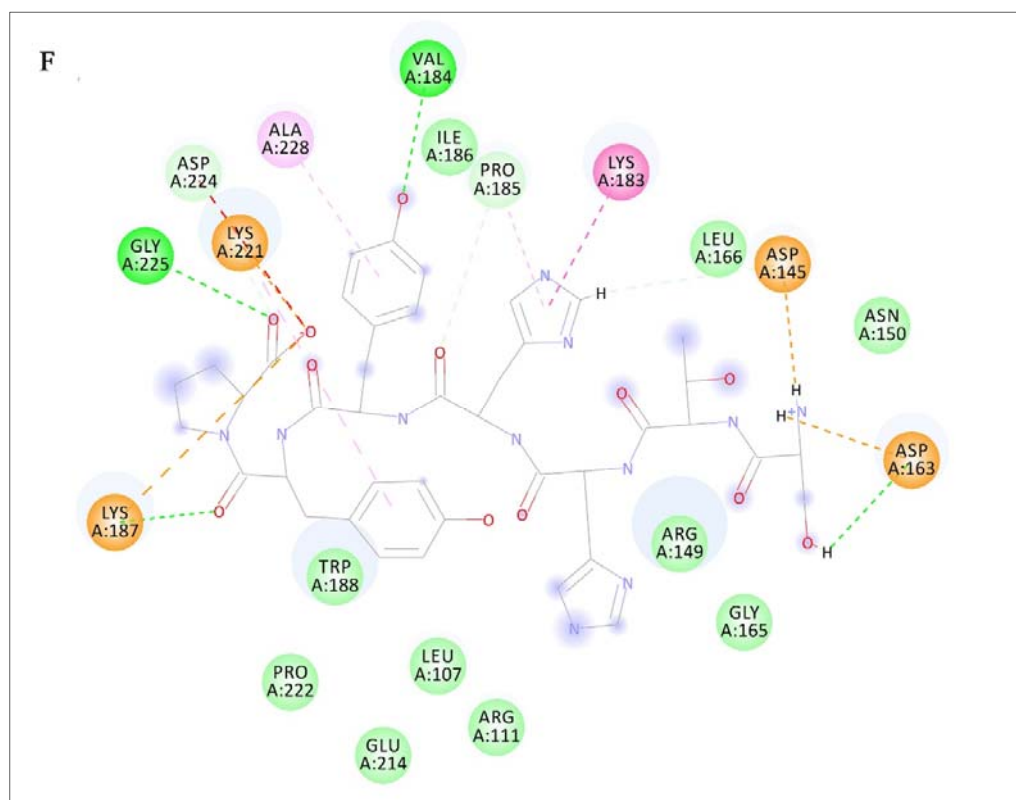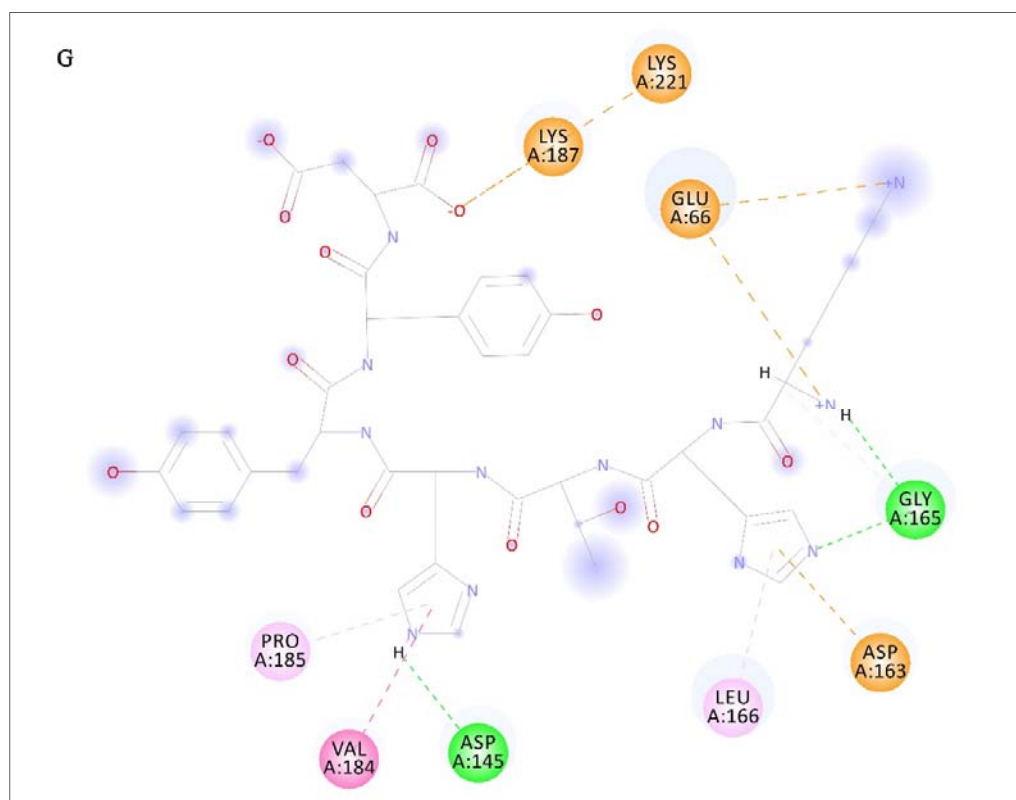

Supplement: S1 Fig — The crystal structure of EGFRL858R-MIG6 pYpY complex (A1), the docked peptides MIG6-YY (B1), peptide 6 (C1), peptide 10 (D1) and peptide 27 (E1) with EGFRL858R. The crystal structure of WT EGFR-MIG6 pYpY complex (A2), the docked peptides MIG6-YY (B2), peptide 6 (C2), peptide 10 (D2) peptide 27 (E2), peptide 5 (F) and peptide 26 (G) with WT-EGFR. (A1) MIG6-pYpY in complex with the crystal structure of EGFRL858R covered only 6 residues of 12 key residues of peptide-substrate binding site, Arg 841, Lys 875, Val 876, Pro 877, Lys 879, Ala 920 and occupied the priming recognition pocket (Lys 879 and Ala 920). Docked peptides MIG6-YY, 6, 10 and 27 (B1, C1, D1 and E1) with EGFRL858R covered 7, 7, 8 and 6 residues of the binding site, respectively and all of them occupied both pockets, phosphoacceptor site (Asp 142) and priming recognition pocket (Lys 184 and Ala 225) except peptide 10) occupied only phosphoacceptor site(. (A2) MIG6-pYpY in complex with the crystal structure of WT-EGFR covered only 5 residues of 12 key residues of peptide-substrate binding site, Lys 875, Val 876, Pro 877, Lys 879, Ala 920 and occupied the priming recognition pocket (Lys 875 and Ala 920). Docked peptides MIG6-YY, 6, 10 and 27 (B2, C2, D2 and E2) with WT-EGFR covered 8, 7, 10 and 8 residues of the binding site, respectively. Docked peptides 5 and 26 (F and G) with WT-EGFR covered 8 and 6 residues of binding site, respectively. (PDF) [file pone.0217031.s001.pdf]
