# Supplementary material for: Structure-based inhibitory peptide design targeting peptide-substrate binding site in EGFR tyrosine kinase
Source: PLoS One. 2019 May 22;14(5):e0217031. doi: 10.1371/journal.pone.0217031 (PMC6530890; doi:10.1371/journal.pone.0217031)
Supplement: S1 Table — (PDF) [file pone.0217031.s006.pdf]

S1 Table: Binding scores of top scoring poses with WT-EGFR and EGFRL858R (PDB: 4ZJV, 4R3R).

Peptide name	Peptide sequences	Interaction scores (REU)		Number of residues*	
		WT-EGFR	EGFR L858R	WT-EGFR	EGFR L858R
MIG6-pYpY	THpYpYLLP	-	-	5	6
MIG6-YY	THYYLLP	-15.695	-16.941	8	7
1	STYYTLP	-15.734	-15.155	5	8
2	TSNGNVLP	-14.598	-18.179	4	7
3	THNNLLP	-15.336	-15.101	8	6
4	TPRHLLP	-16.792	-16.565	6	7
5	STHHYYP	-16.599	-19.640	8	9
6	STHHYYL	-17.608	-19.131	7	7
7	THHYYLP	-16.283	-14.386	9	5
8	THHYYPL	-16.432	-14.874	10	6
9	HTHYYPL	-17.832	-15.561	7	6
10	HTHYYLP	-21.908	-20.212	10	8
11	HSHYYLP	-15.298	-17.939	6	10
12	TSHYYPL	-15.733	-17.238	6	9
13	THYELLP	-15.489	-14.287	8	7
14	STQQLLP	-13.968	-14.273	7	4
15	TTHYYLP	-13.451	-13.624	4	5
16	TTYYHHP	-14.395	-16.913	7	7
17	TTHHYYP	-17.868	-17.766	7	10
18	SSHHYYP	-16.739	-16.278	6	8
19	STHYHYP	-17.390	-16.389	7	5
20	STHHYYP	-15.602	-17.231	6	8
21	SHTHYYF	-18.951	-16.600	9	4
22	SHTHYYD	-14.567	-18.136	6	7
23	STHHYYD	-16.254	-17.543	6	8
24	SHTHYYE	-18.214	-18.720	7	4
25	SHTHYYR	-18.096	-17.710	8	8
26	KHTHYYD	-16.047	-19.982	6	8

27	KRTHYYD	-16.638	-20.093	8	6
28	STHYYCD	-15.955	-15.961	5	7
29	STHYYHD	-16.567	-18.116	6	6
30	HENYYED	-19.879	-15.918	10	7
31	RHDYYWD	-18.840	-18.745	4	6

REU: Rosetta Energy Unit is a standard energy unit used by FlexPepDock server to score complexes

* Indicates the number of amino acids in the EGFR peptide substrate binding site in interaction with the peptides
