# Supplementary material for: Structure-based inhibitory peptide design targeting peptide-substrate binding site in EGFR tyrosine kinase
Source: PLoS One. 2019 May 22;14(5):e0217031. doi: 10.1371/journal.pone.0217031 (PMC6530890; doi:10.1371/journal.pone.0217031)
Supplement: S2 Table — (PDF) [file pone.0217031.s007.pdf]

S2 Table. Comparison of WT-EGFR and EGFRL858R total binding energy calculated by Rosetta FlexPepDock and MM-PBSA.

		Rosetta FlexPepDock Server (REU)		MM-PBSA (kcal/mol)	
**Peptides**	**Peptide sequences**	**WT-EGFR**	**EGFR L858R**	**WT-EGFR**	**EGFR L858R**
**MIG6-YY**	THYYLLP	-15.695	-16.941	-16.397	-18.706
**Peptide 5**	STHHYYP	-16.599	-19.640	-4.497	-23.923
**Peptide 6**	STHHYYL	-17.608	-19.131	-30.592	-9.205
**Peptide 10**	HTHYYLP	-21.908	-20.212	-15.675	-44.791
**Peptide 26**	KHTHYYD	-16.047	-19.982	-8.544	-25.098
**Peptide 27**	KRTHYYD	-16.638	-20.093	-45.258	-46.426
