# Supplementary material for: Structure-based inhibitory peptide design targeting peptide-substrate binding site in EGFR tyrosine kinase
Source: PLoS One. 2019 May 22;14(5):e0217031. doi: 10.1371/journal.pone.0217031 (PMC6530890; doi:10.1371/journal.pone.0217031)
Supplement: S3 Table — (PDF) [file pone.0217031.s008.pdf]

S3 Table. **Physicochemical properties of peptides MIG6-pYpY, MIG6-YY, 5, 6, 10, 26 and 27.**

Peptides	MW (Da)	HBD	HBA	LogP	LogD	LogSw	Solubility (mg/l)
**MIG6-pYpY**	1053.02	16	25	-0.37	-3.62	-4.44	12378.22
**MIG6-YY**	908.05	13	20	1.56	-0.89	-5.03	5928.75
**5**	905.95	15	23	-2.37	-5.24	-2.65	64168.61
**6**	904.99	15	22	-0.84	-3.44	-3.40	30079.38
**10**	915.03	13	21	0.52	-2.20	-4.45	10665.21
**26**	955.07	18	24	-4.56	-8.00	-1.11	315731.47
**27**	974.11	22	25	-5.03	-8.47	-0.73	470356.39
